# Label-free quantitative proteomic analysis of gingival crevicular fluid to identify potential early markers for root resorption

**DOI:** 10.1186/s12903-020-01246-9

**Published:** 2020-09-11

**Authors:** Farah Amirah Mohd Nasri, Shahrul Hisham Zainal Ariffin, Saiful Anuar Karsani, Rohaya Megat Abdul Wahab

**Affiliations:** 1 Department of Biological Sciences and Biotechnology, Faculty of Science and Technology, Universiti Kebangsaan Malaysia, 43600 Bangi, Selangor Malaysia; 2 Malaysia Genome Institute (MGI), National Institute of Biotechnology Malaysia (NIBM), Jalan Bangi, 43000 Kajang, Selangor Malaysia; 3 Institute of Biological Sciences, Faculty of Science, Universiti Malaya, 50603 Kuala Lumpur, Malaysia; 4 Department of Family Oral Health, Faculty of Dentistry, Universiti Kebangsaan Malaysia, Jalan Raja Muda Abdul Aziz, 50300 Kuala Lumpur, Malaysia

**Keywords:** Root resorption, Early biomarker, Gingival crevicular fluid, Proteomics, Orthodontic treatment

## Abstract

**Background:**

Orthodontically-induced root resorption is an iatrogenic effect and it cannot be examined regularly due to the harmful effects of sequential doses of radiation with more frequent radiography. This study aims to compare protein abundance (PA) of pre-treatment and during orthodontic treatment for root resorption and to determine potential early markers for root resorption.

**Methods:**

Ten subjects (*n* = 10) who had upper and lower fixed appliances (MBT, 3 M Unitek, 0.022″ × 0.028″) were recruited for this study. Human gingival crevicular fluid (GCF) was obtained using periopaper strips at pre-treatment (T0), 1 month (T1), 3 months (T3), and 6 months (T6) of orthodontic treatment. Periapical radiographs of the upper permanent central incisors were taken at T0 and T6 to measure the amount of root resorption. Identification of changes in PA was performed using liquid chromatography-tandem mass spectrometry. Student’s t-test was then performed to determine the significance of the differences in protein abundance before and after orthodontic treatment.

**Results:**

Our findings showed that all ten subjects had mild root resorption, with an average resorption length of 0.56 ± 0.30 mm. A total of 186 proteins were found to be commonly present at T0, T1, T3, and T6. There were significant changes in the abundance of 16 proteins (student’s t-test, *p* ≤ 0.05). The increased PA of S100A9, immunoglobulin J chain, heat shock protein 1A, immunoglobulin heavy variable 4–34 and vitronectin at T1 suggested a response to stress that involved inflammation during the early phase of orthodontic treatment. On the other hand, the increased PA of thymidine phosphorylase at T3 suggested growth promotion and, angiogenic and chemotactic activities.

**Conclusions:**

The identified proteins can be potential early markers for root resorption based on the increase in their respective PA and predicted roles during the early phase of orthodontic treatment. Non-invasive detection of root resorption using protein markers as early as possible is extremely important as it can aid orthodontists in successful orthodontic treatment.

## Background

Orthodontic treatment has iatrogenic effects that can be detrimental to the stability of teeth and shorten their roots. Root resorption occurs when the root is shortened, which often results in tooth extraction (in severe cases) [1]. Orthodontic treatment applies a mechanical force [2] that is capable of creating external root resorption in the patients, hence shortening the roots [3, 4]. Gingival crevicular fluid (GCF) is an oral cavity fluid and its content may be representative of the body’s health [5–7]. GCF contains proteins involved in oral pathologic phenomenon, such as internal root resorption [8, 9], as well as a diverse population of cells, desquamated epithelial cells, organic ions, inorganic ions, enzymes, and bacteria from adjacent plaque. GCF has attracted researchers for its potential use as a diagnostic fluid for periodontal diseases and drug analyses due to its non-invasive sampling [8–10]. As such, we believe that GCF is a reliable source for root resorption biomarkers.

Radiography is currently the primary approach in determining root resorption. A radiograph is an image produced on a sensitive plate or film by X-rays, gamma rays, or similar radiation [11, 12]. Frequent radiographs may have negative effects on patients [1, 13, 14]. As radiography cannot be conducted regularly, it limits its utility as a tool for monitoring a patient’s teeth. Therefore, identification and the subsequent utilization of biomarkers for root resorption would be a useful and attractive alternative to monitoring root resorption. A non-invasive way to determine the severity of root resorption is by detecting the presence of protein biomarkers in GCF. Comparative proteomics using liquid chromatography-mass spectrometry (LC-MS) can be used to screen and compare GCF proteome profiles and identify potential root resorption biomarkers. This study is a preliminary investigation to identify potential diagnostic biomarkers for root resorption during the early phase of orthodontic treatment. Eventually, in practice, detection of the identified biomarkers will be performed using protein assay or enzyme-linked immunosorbent assay (ELISA) technique. These biomarkers will allow continuous monitoring of root resorption and better management of the condition. Our study aims to compare the protein abundance (PA) of GCF at pre-treatment (T0), after 1 month (T1), 3 months (T3), and 6 months (T6) of orthodontic treatment. This is will allow us to identify potential early root resorption biomarkers.

## Methods

### Subjects

Subjects were patients (*n* = 10) who have undergone treatment at the Postgraduate Orthodontic Clinic 1, Faculty of Dentistry, Universiti Kebangsaan Malaysia, Kuala Lumpur. All wore upper and lower fixed appliances (MBT, 3 M Unitek, 0.022″ × 0.028″). The exclusion criteria were patients who were pregnant, smokers, who had previous orthodontic treatment, a non-vital tooth, a treated root or fractured root, and patients who had consumed anti-inflammatory drugs in the month preceding the study.

### Ethical approval and sample collection

This study was approved by the Ethics Research Committee, Universiti Kebangsaan Malaysia (UKM PPI/111/8/JEP-2018-268), and patients were briefed and written informed consent was obtained. GCF sampling was carried out using Periopaper strips (Proflow, USA). Strips were inserted at the gingival sulcus mesial and distal side of the upper permanent central incisor at approximately 1–2 mm of gingiva depth for 60 s. This was repeated three times with intervals of 60 s in between. Each crevicular sulcus on the test tooth was dried with cotton rolls, and a saliva ejector was used to remove the remaining saliva prior to insertion of the periopaper strip. Sampling was conducted at T0, T1, T3, and T6. The periopaper strips were then placed into microcentrifuge tubes containing protease inhibitors.

### Radiography

Periapical radiographs of the upper permanent central incisor were taken at T0 and T6 to measure the amount of root resorption by the same radiographer.

### Extraction and digestion of proteins

GCF samples were centrifuged at 10,000×*g* for 15 min at 4 °C and proteins were extracted. Protein content was estimated by Bradford Assay. Ammonium bicarbonate (50 mM) and dithiothreitol (100 mM) were put into 1.5 mL microcentrifuge tubes. A total of 100 ng of the GCF protein was then added to the tubes, and incubated at 95 °C for 5 min. The mixtures were allowed to cool at room temperature for 5 min. Iodoacetamide (100 mM) was added to the tubes and incubated in the dark at room temperature for 20 min. Trypsin (100 ng/μL) was then pipetted into the reaction tube and incubated at 37 °C for 3 h, followed by overnight incubation at 30 °C. Digested samples were dried using a SCANVAC Vacuum Concentrator (LaboGene, Denmark) at low speed. The samples were then dissolved in 0.1% formic acid in the reaction tubes and with vigorous vortexing.

### Separation of digested peptides

Digested peptides were resolved using a Dionex UltiMate™ 3000 RSLCnano system (Thermo Fisher Scientific, USA) with an EASY-Spray Column Acclaim PepMap™ C18 100 A0 separation column, a 2 μm particle size, and a 50 μm internal diameter with 15 cm in length. The flow rate was 250 nL/min and mobile phase A and B contained 0.1% formic acid in water and 0.1% formic acid in acetonitrile, respectively. The gradient was the following: 5–40% mobile phase B for 91 min, 85% mobile phase B for 2 min, 85% mobile phase B for 3 min, 5% mobile phase B for 1 min and 5% mobile phase B for 4 min. The column temperature was maintained at 35 °C.

### Mass spectrometry data acquisition

The mass spectrometric analysis was conducted using the Orbitrap Fusion Mass Spectrometer (Thermo Fisher Scientific, USA) in data-dependent mode. Full scan spectra were collected with orbitrap MS (OTMS) using the following parameters: scan range, 310–1800 m/z; resolving power of 120,000; automatic gain control (AGC) target of 4.0e5 (400,000); and maximum injection time of 50 ms. The method consisted of 3 s of top speed mode where the precursors were selected for a maximum of 3 s cycles. Only precursors with an assigned monoisotopic m/z and a charge state of 2–7 were further analysed for second stage mass spectrometry (MS2). All precursors were filtered using a 20 s dynamic exclusion window with intensity threshold of 5000. The MS2 spectra were analysed by ion trap mass spectrometry (ITMS) using the following parameters: rapid scan rate with a resolving power of 60,000, AGC target of 1.0e2 (100), 1.6 m/z isolation window, and a maximum injection time of 250 ms.

### Protein identification

Proteins were identified using the Thermo Scientific™ Proteome Discoverer Software Version 2.1 where the mass spectra were searched against the *Homo sapiens* UniProt database [15]. All peptides were validated using the percolator® algorithm based on a q-value of less than a 5% false discovery rate (FDR). Following the identification of proteins, PANTHER software (Protein analysis through evolutionary relationships) was used to predict the biological processes affected during orthodontic treatment [16]. Search Tools or the Retrieval of Interesting Proteins/Genes (STRING) version 11.0 was then utilized to determine pathways involved during orthodontic treatment [17].

### Statistical analysis

All analyses were performed using Microsoft Excel. The intra-examiner correlation coefficient (ICC) was calculated to determine the reliability of the measurements for the amount of root resorption at 6 months. Student’s t-test was used to determine the statistical signficance in PA during the first 6 months of orthodontic treatment. A difference was considered significant when *p* ≤ 0.05.

## Results

The differences in the root length ranged from 0.14–1.01 mm, while the crown length ranged from 0.15–0.91 mm (Table 1). The average difference in root resorption was 0.56 ± 0.30 mm, whereas the average difference in crown length was 0.37 ± 0.23 mm. Based on the radiographs, all subjects were shown to be having mild root resorption (< 2 mm). Since all subjects have similar root resorption rates, the samples were chosen randomly based on patient’s availability.

**Table 1:** Demographic, crown length and root length of orthodontic patients. C1 and R1 refer to the tooth length prior to orthodontic treatment, while C2 and R2 were taken after 6 months of orthodontic treatment. Subjects that were chosen labelled as*

Subjects	Age	Crown length; C1 (mm)	Crown length; C2 (mm)	Difference of Crown length C1-C2 (mm)	Root length; R1 (mm)	Root length; R2) (mm)	Difference of Root length R1-R2 (mm)
1	25	9.37 ± 0.43	9.08 ± 0.20	0.29	17.85 ± 0.12	17.50 ± 0.02	0.35
2	24	8.14 ± 0.07	7.89 ± 0.06	0.25	15.15 ± 0.17	14.71 ± 0.12	0.44
3	17	9.18 ± 0.26	8.96 ± 0.20	0.22	15.90 ± 0.29	15.28 ± 0.10	0.62
4*	25	10.75 ± 0.66	10.23 ± 0.41	0.52	18.17 ± 0.36	17.30 ± 0.57	0.87
5	24	9.60 ± 0.63	9.33 ± 0.70	0.27	16.59 ± 0.49	16.15 ± 0.56	0.44
6*	24	9.65 ± 0.15	9.50 ± 0.23	0.15	19.71 ± 0.11	19.57 ± 0.35	0.14
7	15	9.33 ± 0.13	9.13 ± 0.04	0.20	16.08 ± 0.04	15.66 ± 0.02	0.42
8	16	9.36 ± 0.31	9.03 ± 0.53	0.33	20.43 ± 0.08	19.43 ± 0.18	1.01
9	17	11.74 ± 0.29	10.83 ± 0.97	0.91	18.58 ± 0.11	17.60 ± 0.87	0.98
10*	19	9.93 ± 0.10	9.39 ± 0.76	0.54	14.54 ± 1.06	14.18 ± 0.84	0.36

**Fig. 1:**
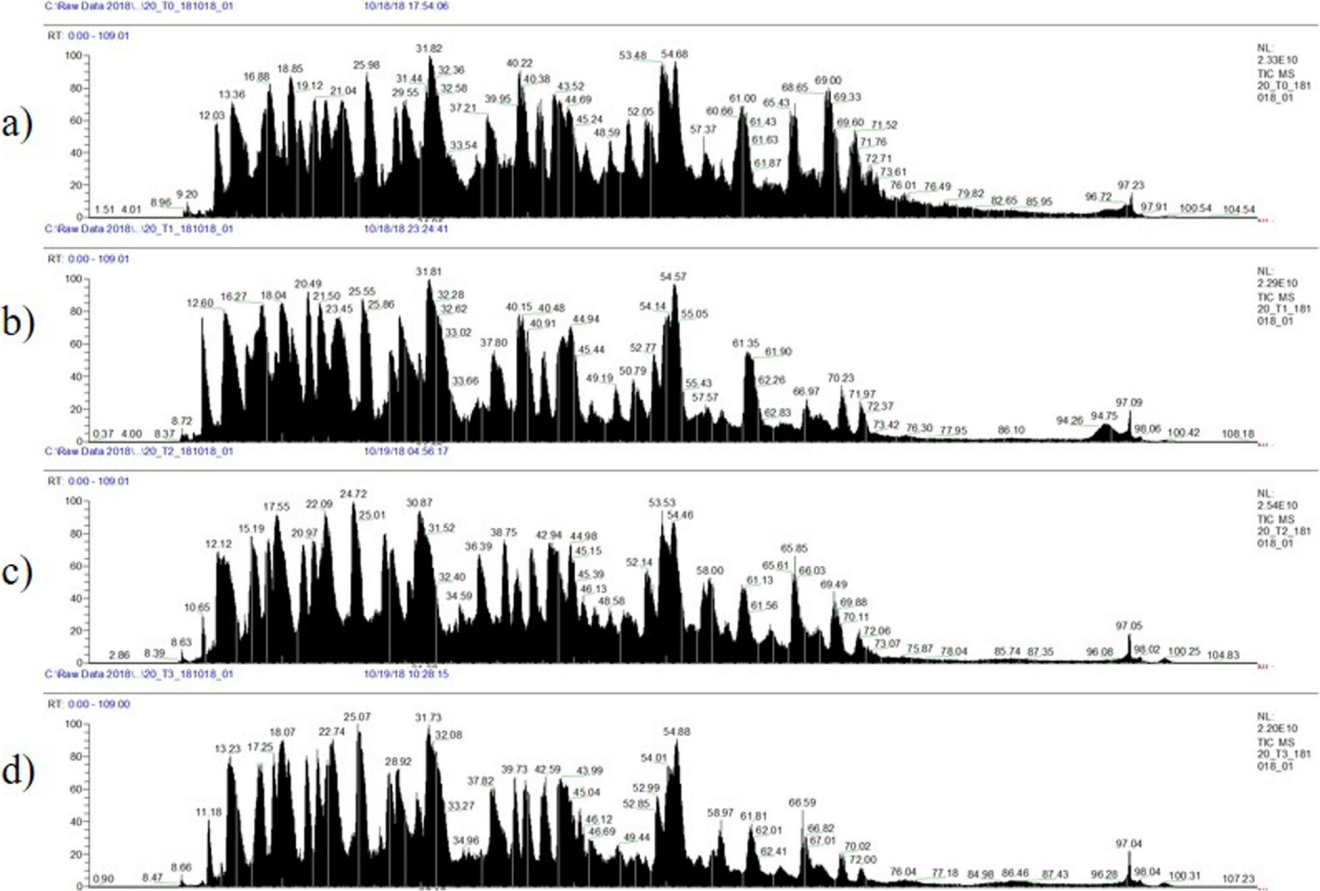
Total ion current (TIC) chromatograms from three subjects (*n* = 3) with total of 12 samples and 3 injections/sample of human gingival crevicular fluid (GCF). **a** Prior to treatment (T0), **b** after 1 month of treatment (T1), **c** after 3 months of treatment (T3), and (**d**) after 6 months of treatment (T6). The graph shows the total intensity versus time (min). Two microlitres of the one microgram per microlitre sample was loaded for 110 min, with a flow rate of 250 nL/min in 0.1% mobile phase B (0.1% formic acid in acetonitrile)

**Fig. 2:**
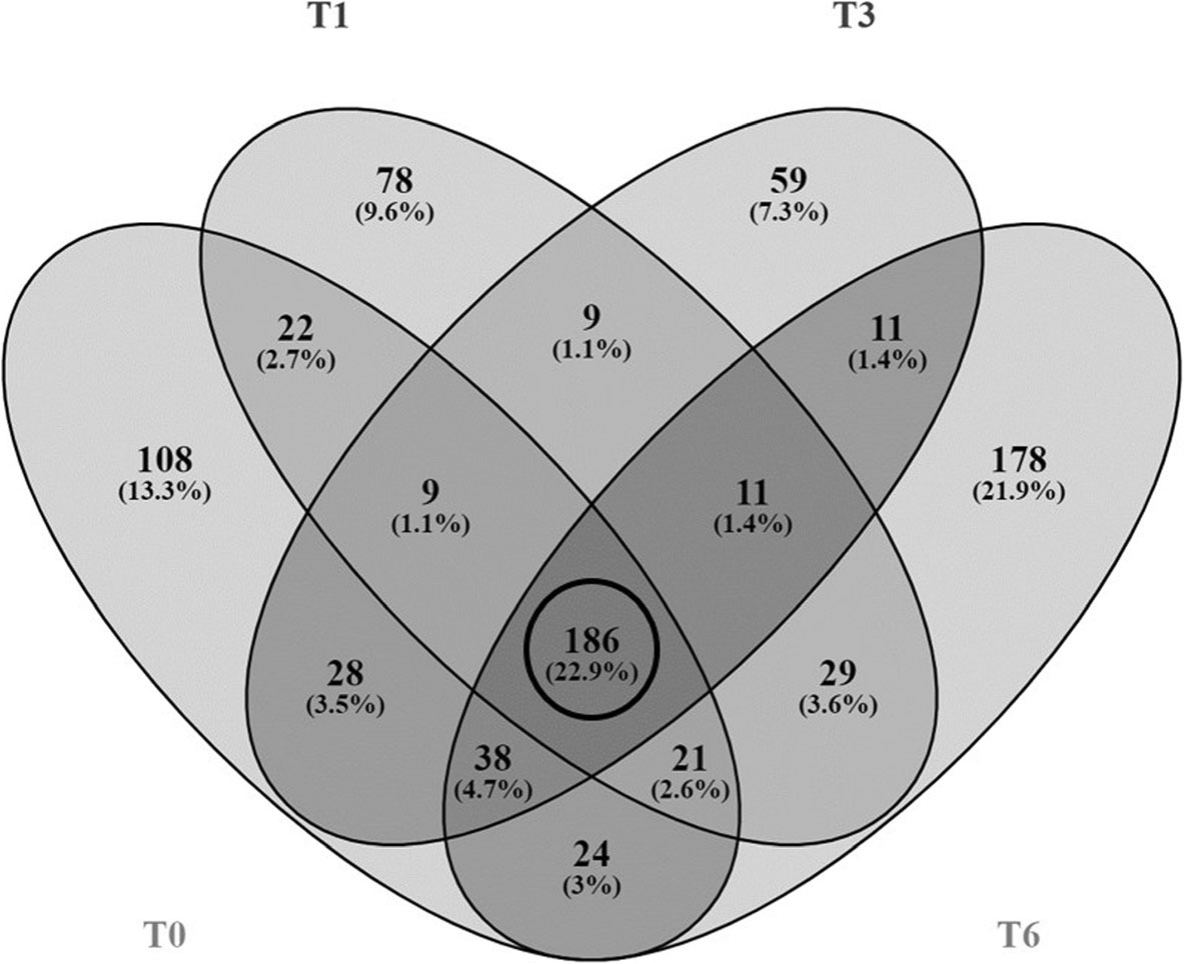
Venn diagram of a number of proteins found in the GCF of 3 individual subjects before orthodontic treatment (T0), after 1 month of treatment (T1), after 3 months of treatment (T3), and after 6 months of treatment (T6). The total protein counts at T0, T1, T3, and T6 were 436, 365, 351, and 498, respectively. One hundred eighty-six proteins (circled)-- were commonly found in all samples

Meanwhile, Fig. 1 shows the base-peak chromatogram for nano-LC monitored by mass spectrometry, representing the intensity of all peptide ions in the sample in a single scan. The GCF proteome from T0 to T6 showed consistent elution of proteins/peptides, ranging from 8 to 100 min. A total of 815 proteins were identified by LC-MS in human GCF. The total proteins at T0, T1, T3, and T6 were 439, 368, 354, and 502, respectively. Furthermore, 186 proteins were commonly found in all samples (Fig. 2). Following statistical analysis, 16 proteins were found to have changed significantly (Student’s t-test, *p* ≤ 0.05). They were S100A9, immunoglobulin J chain (IGJ), angiotensinogen (AGT), apolipoprotein D (APOD), beta-2-glycoprotein 1 (APOH), hemoglobin subunit beta (HBB), histidine-rich glycoprotein (HRG), immunoglobulin heavy variable 3–11 (IGHV3–11), immunoglobulin heavy constant gamma 1 (IGHG1), thymosin beta-4 (TMSB4X), zinc-alpha-2-glycoprotein (AZGP1), heat shock protein 1A (HSPA1A) (70 kDa), immunoglobulin heavy variable 4–34 (IGHV4–34), thymidine phosphorylase (TYMP), immunoglobulin kappa variable 3–20 (IGKV3–20), and vitronectin (VTN) (Table 2).

**Table 2:** Biological process, protein class, and pathway of significant proteins. Cellular process (CP), metabolic process (MP), immune system process (ISP), localisation (L), response to stimulus (RS), and biological regulation (BR). The proteins found to be involved in the biological processes are represented with symbol X

Protein name	Gene name	Accession no (SwissProt)	Biological process						Protein class
			CP	MP	ISP	L	RS	BR	
Angiotensinogen	AGT	P01019	**X**						Enzyme modulator
Apolipoprotein D	APOD	P05090		**X**			**X**		–
Beta-2-glycoprotein 1	APOH	P02749							–
Heat shock 70 kDa protein 1A	HSPA1A	P0DMV8	**X**	**X**		**X**	**X**	**X**	–
Hemoglobin subunit beta	HBB	P68871	**X**				**X**		–
Histidine-rich glycoprotein	HRG	P04196	**X**			**X**	**X**	**X**	Enzyme modulator
Ig heavy variable 4–34	IGHV4–34	P06331	**X**	**X**	**X**	**X**	**X**	**X**	–
Ig kappa variable 3–20	IGKV3–20	P01619			**X**				Defense or immunity protein
Ig heavy variable 3–11	IGHV3–11	P01762	**X**	**X**	**X**	**X**	**X**	**X**	–
Ig heavy constant gamma 1	IGHG1	P01857	**X**	**X**	**X**	**X**	**X**	**X**	–
Ig J chain	IGJ	P01591							–
Thymidine phosphorylase	TYMP	P19971							Transferase
Protein S100-A9	S100A9	P06702	**X**	**X**					Calcium-binding protein, signaling molecule
Thymosin beta-4	TMSB4X	P62328	**X**			**X**		**X**	–
Zinc-alpha-2-glycoprotein	AZGP1	P25311			**X**			**X**	–
Vitronectin	VTN	P04004	**X**						–

**Fig. 3:**
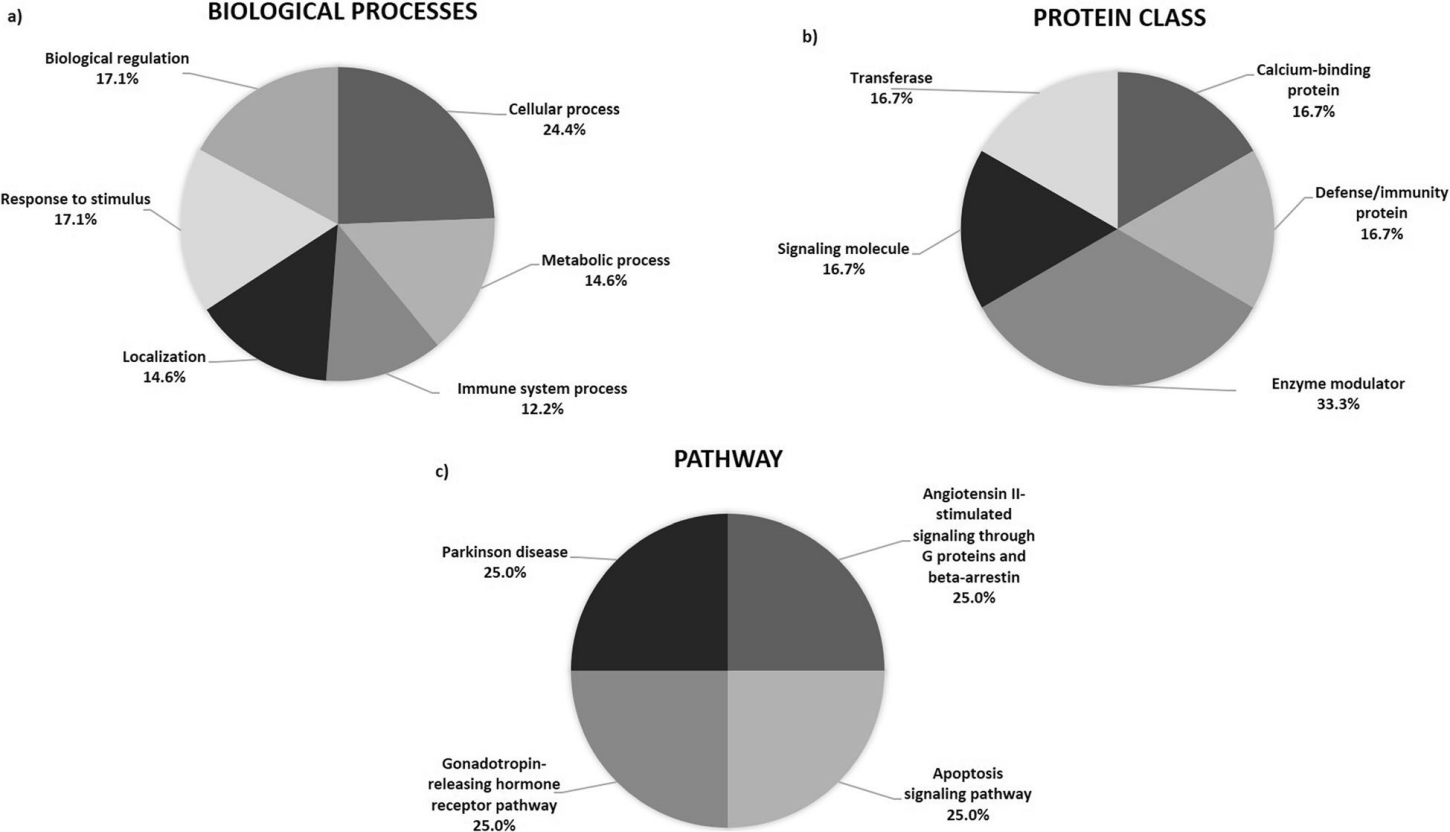
Pie chart representing the; **a**) biological process, **b**) protein class, and **c**) pathway of the proteins that changed significantly based on Protein Analysis Through Evolutionary Relationships (PANTHER) database. The biological processes involved were cellular processes, metabolic processes, immune system processes, localisation, responses to stimuli, and biological regulation. The protein classes involved were calcium-binding proteins, defence or immunity proteins, enzyme modulators, signalling molecules, and transferase. While, the identified proteins also involved in other pathways were angiotensin II-stimulated signalling through G proteins and beta-arrestin, the apoptosis signalling pathway, the gonadotropin-releasing hormone receptor pathway, and Parkinson pathway

These proteins were then analysed using the Protein Analysis Through Evolutionary Relationships (PANTHER, http://pantherdb.org) database. This led to the identification of six biological processes [18] that may be associated with these changes. Of the proteins, 24.4% changed significantly and they were involved in cellular processes (Fig. 3a). Other biological processes (more than 10%) were associated with metabolic processes, immune system processes, localisation, response to stimuli, and biological regulation, as shown in Fig. 3a. PANTHER found five protein classes that were involving during treatment (Fig. 3b). They were a calcium-binding proteins, defence or immunity proteins, enzyme modulators, signalling molecules, and transferases [18]. Two proteins, AGT and HRG, out of sixteen proteins were enzyme modulators. The SEQUEST score, pI/MW, peptide matched (% sequence coverage), fold change, and *p*-value of significant proteins are shown in Table 3.

**Table 3:** Differentially expressed proteins in GCF at T1, T3, and T6 during orthodontic treatment when compared to T0 (pre-treatment). There was a change in the expression of 16 proteins in the GCF. Increased and decreased protein abundance (PA) of significant proteins are represented in the form of ↑ and ↓ (Student’s T-test, *p*-value ≤0.05)

Protein name	Gene name	Accession number (SwissProt)	pI/MW (kDa)	Sequest score	Peptide matched (% sequence coverage)	Fold change; differential protein abundance (↑/↓)			Student’s t-test; *p*-value
						T1	T3	T6	
Angiotensinogen	AGT	P01019	6.32/53.12	2.21	2.47	1.03↓	1.04↓	0.98↓	0.03
Apolipoprotein D	APOD	P05090	5.15/21.26	2.11	5.82	1.20↓	1.13↓	0.99↓	0.05
Beta-2-glycoprotein 1	APOH	P02749	7.97/38.27	171.74	35.36	1.18↓	1.10↓	1.12↓	0.02
Heat shock 70 kDa protein 1A	HSPA1A	P0DMV8	5.66/70.01	96.86	12.79	1.14↓	0.99↑	1.02↑	0.01
Hemoglobin subunit beta	HBB	P68871	7.28/15.99	429.06	89.80	1.15↓	1.14↓	1.07↓	0.03
Histidine-rich glycoprotein	HRG	P04196	7.5/59.54	12.46	2.67	1.13↓	1.05↓	1.18↓	0.02
Ig heavy variable 4–34	IGHV4–34	P06331	8.28/16.22	16.36	10.96	1.02↓	0.99↑	1.01↑	0.03
Ig kappa variable 3–20	IGKV3–20	P01619	8.48/11.77	187.22	31.19	1.11↓	1.08↓	1.01↑	0.03
Ig heavy variable 3–11	IGHV3–11	P01762	9.72/13.46	19.17	15.57	1.08↓	1.03↓	1.12↓	0.04
Ig heavy constant gamma 1	IGHG1	P01857	8.19/36.08	848.34	51.52	1.12↓	1.07↓	0.99↓	0.03
Ig J chain	IGJ	P01591	5.24/18.09	15.71	16.35	0.98↑	0.98↑	1.05↑	0.03
Thymidine phosphorylase	TYMP	P19971	5.53/50.37	14.68	2.87	1.07↓	0.97↑	0.83↓	0.03
Protein S100-A9	S100A9	P06702	6.13/13.23	44.48	24.56	0.97↑	0.95↑	1.07↑	0.02
Thymosin beta-4	TMSB4X	P62328	5.06/5.05	143.02	54.55	1.02↓	1.00↓	1.04↓	0.05
Zinc-alpha-2-glycoprotein	AZGP1	P25311	6.05/34.24	108.53	19.46	0.95↑	0.90↑	1.13↑	0.04
Vitronectin	VTN	P04004	5.8/54.27	239.34	23.85	0.99↑	1.00↓	1.064↑	0.04

**Fig. 4:**
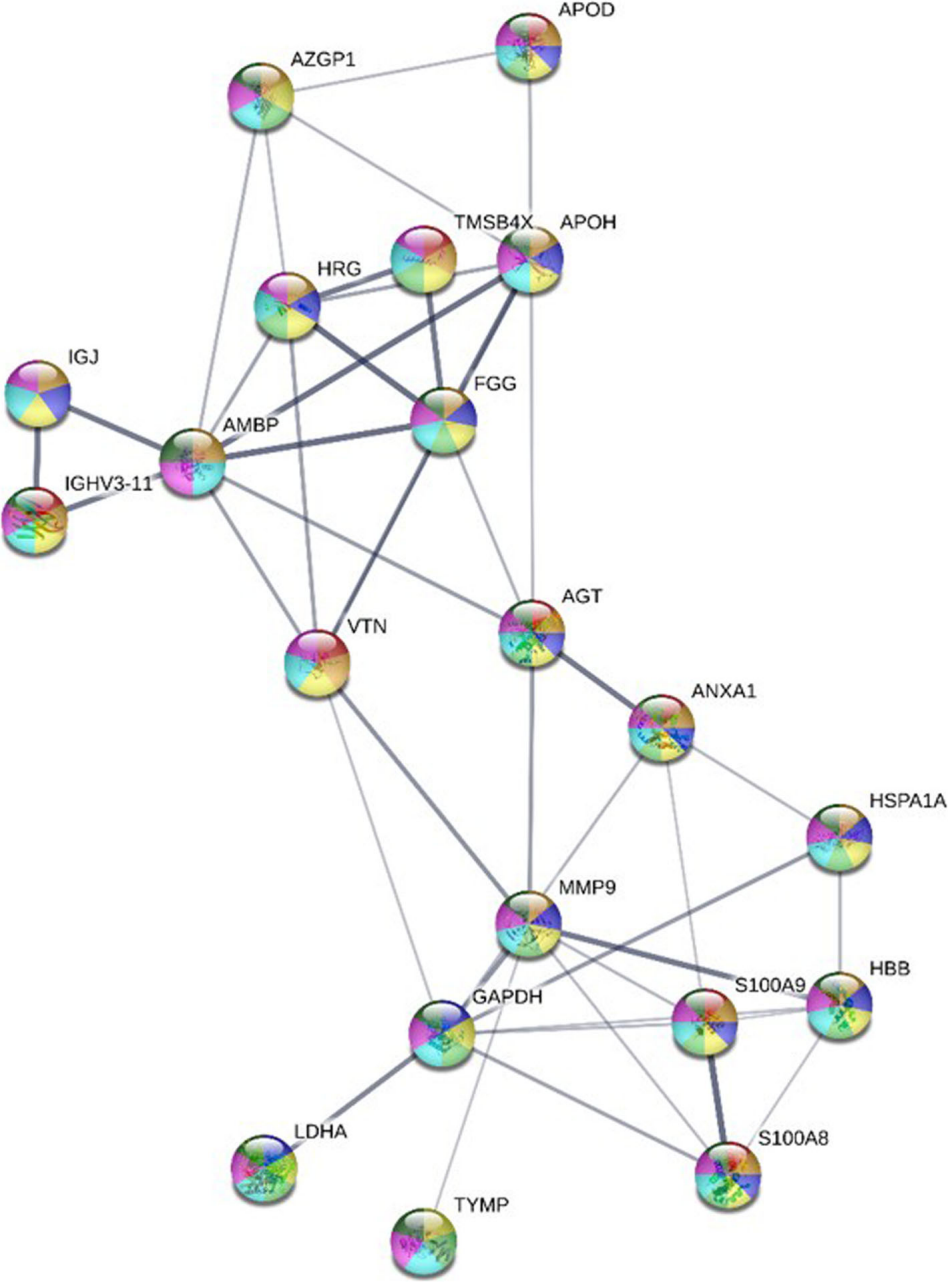
Protein interaction networks of proteins that significantly changed in abundance, determined using STRING version 11.0. A total of 13 proteins were analysed. Seven additional proteins were added to determine protein interactions during orthodontic treatment. Each protein was represented with a node and its respective gene name. The colours of the node representations are as follows: red, positive regulation of biological process; dark green, response to stimulus; purple, metabolic process; dark blue, cellular process; light green, immune system process; and yellow, regulation of localisation. Meanwhile, interactions of proteins were identified using the edges connecting the nodes. The thickness of the edge is represented as follows: thinnest, low confidence (interaction score: 0.150); thin, medium confidence (interaction score: 0.400); thick, high confidence (interaction score: 0.700); and thickest, highest confidence (interaction score: 0.900)

S100A9 is a calcium-binding protein and a signalling molecule. The identified proteins were also involved in other pathways, as suggested by PANTHER, and these were the angiotensinogen II-stimulated signalling through G proteins and β-arrestin [19], the apoptosis signalling pathway [18], the gonadotropin-releasing hormone receptor pathway [20], and Parkinson pathway [21] (Fig. 3c). Proteins that were significantly different in abundance were also analysed using STRING analysis. Thirteen proteins were predicted to be associated with six pathways (Table 4). The interactions between these proteins are shown in Fig. 4.

**Table 4:** Protein interaction network found in the GCF with an additional seven proteins in the Search Tools or the Retrieval of Interesting Proteins/Genes (STRING) analysis. A total of six pathways were found during orthodontic treatment

Pathway id	Pathway description	Observed Gene count	False discovery rate	Nodes colour	Associated proteins
GO:0048518	Positive regulation of biological process	16	4.31E-05	Red	AGT, ANXA1, APOH, FGG, GAPDH, HBB, HRG, HSPA1A, IGHV3–11, IGJ, LDHA, MMP9, S100A8, S100A9, TMSB4X, VTN
GO:0050896	Response to stimulus	19	1.10E-05	Dark green	AGT, ANXA1, APOD, APOH, AZGP1, FGG, GAPDH, HBB, HRG, HSPA1A, IGHV3–11, IGJ, LDHA, MMP9, S100A8, S100A9, TMSB4X, TYMP, VTN
GO:0008152	Metabolic process	16	0.0178	Purple	AGT, AMBP, ANXA1, APOD, APOH, AZGP1, FGG, GAPDH, HBB, HSPA1A, IGHV3–11, LDHA, MMP9, S100A8, S100A9, TYMP
GO:0009987	Cellular process	20	0.0134	Dark blue	AGT, AMBP, ANXA1, APOD, APOH, AZGP1, FGG, GAPDH, HBB, HRG, HSPA1A, IGHV3–11, IGJ, LDHA, MMP9, S100A8, S100A9, TMSB4X, TYMP, VTN
GO:0002376	Immune system process	12	1.42E-05	Light green	ANXA1, FGG, GAPDH, HBB, HRG, HSPA1A, IGHV3–11, IGJ, MMP9, S100A8, S100A9, VTN
GO:0032879	Regulation of localization	13	4.39E-06	Yellow	AGT, ANXA1, APOD, APOH, FGG, GAPDH, HRG, HSPA1A, MMP9, S100A8, S100A9, TMSB4X, VTN

## Discussion

### Radiography

Root resorption is an unavoidable consequence of orthodontic treatment. At present, root resorption can only be detected using radiographs. Periapical radiographs have previously been used to measure root resorption [1, 14, 22]. In this study, periapical radiographs were obtained on central incisors at T0 and T6 as evidence of root resorption. The roots and crowns of all subjects were measured three times. Although radiography is the gold standard for measurement of root resorption, it has limitations. Radiation exposure due to frequent radiography procedures can be detrimental to humans and may lead to radiation carcinogenesis [23]. The dosage of radiation should be as low as rationally obtainable, and the number of radiographs should be chosen wisely because the risk will increase as the frequency of X-ray exposure increases [23]. In this study, a periapical radiograph was obtained twice, before treatment and at 6 months of orthodontic treatment, and this was found to be acceptable [24].

### Root resorption

All subjects were shown to have mild root resorption, most likely as they were in the early stage of orthodontic treatment (first 6 months). This has been previously observed in other reports (< 2 mm at sixth months of orthodontic treatment) [25]. GCF samples from the first sixth months of orthodontic treatment is therefore a useful source for the identification of proteins involved in root resorption. Prolonged orthodontic treatment may deteriorate the root, potentially causing severe root resorption [26]. It has been shown that severe root resorption (≥ 2 mm) occured in patients after 12 months of orthodontic treatment [25]. The long duration of orthodontic treatment caused the teeth to be exposed to a long-term jiggling force, causing an increase in root resorption [27]. In this study, we targeted proteins from the early phase of orthodontic treatment that can be useful root resorption early markers. Once identified and confirmed, in practice, detection of the identified biomarkers will be conducted using protein assay or ELISA techniques. When a certain threshold or panel of protein biomarkers have been detected, orthodontic force can be lowered or treatment can be stopped to prevent severe root resorption.

### Gingival crevicular fluid (GCF) biomarker

To date, there are no reliable biomarkers from GCF that can be used in measuring root resorption during orthodontic treatment. Previous studies have been focused on identifying biomarkers related to periodontal disease, systemic diseases, and pubertal growth [28–31]. Furthermore, other previous studies used non-resorbing and resorbing deciduous molars to identify unique root resorption biomarkers [32].

Due to its ability to be site-specific, GCF can be an unparalleled source for identifying specific dental biomarkers of oral conditions [33]. GCF is known to be unique and different from saliva [34, 35]. Many researchers have classified GCF as an inflammatory exudate, and some have suggested that it is a transformed tissue transudate in a normal healthy state [7, 34, 36, 37], which consists of proteins, a distinct population of cells, exfoliated epithelial cells, and bacteria from neighbouring plaque [5]. In this study, we compared samples from T0, which is control (normal) to samples at T1, T3 and T6. T0 samples were acquired before subjects began wearing braces.

### Proteomics

Proteomics has many advantages in the identification of potential disease biomarkers as it can detect low levels of a specific proteins [38]. In relation to our work, a biomarker can be used to detect the severity of root resorption. In this study, we used proteomics to determine the protein profile of GCF samples before treatment and at 6 months of orthodontic treatment. Previous studies have also used a proteomics approach to identify disease biomarkers [39, 40]. A separate study used proteomics to identify the protein expression of bacteria [41].

### Potential early markers for root resorption

Table 3 shows that both S100A9 and IGJ had increased PA from T0 to T6. S100A9 is a calcium- and zinc-binding protein that plays a prominent role in the regulation of inflammatory processes and immune responses. It can induce neutrophil chemotaxis and adhesion, and it can increase the bactericidal activity of neutrophils by promoting phagocytosis [42, 43]. However, this protein was reported to be downregulated on day 14 compared to day 0 of orthodontic treatment [44]. This suggested that activation of inflammation occurred after 1 month as this protein has known roles in inflammation and bone resorption [44]. Another study stated that S100A9 was distinguished in osteoclasts and was involved in chondrocyte and osteoblast maturation [43].

IGJ (J Chain) has a prominent role in the binding immunoglobulin units - IgM or IgA [45]. It can induce the formation of larger polymers when binding to IgA. It also helps to bind immunoglobulins to secretory component [44]. A previous study have found significant differences in the mean serum levels of IgA, IgG, and IgM between well-controlled type 1 diabetic patients that wore orthodontic appliances and those who did not [46]. IGJ has been found to be involved in the inflammatory process and immune response during the early phase of orthodontic treatment. A separate study found increased expression of IGJ in type 2 diabetes mellitus patients with periodontitis compared to controls [47]. Periodontitis is a condition where the periodontium is inflamed, mainly induced by a bacterial infection [9]. This may provide an explanation for the high level of IGJ during the inflammatory process. Nonetheless, a past study reported that IGJ was downregulated after 14 days of orthodontic treatment [44]. This was likely due to the inflammation process not being active on day 14 of orthodontic treatment [48].

Conversely, AGT, APOD, APOH, HBB, HRG, IGHV3–11, IGHG1, TMSB4X, and AZGP1 showed decreased PA in all samples. A decrease in PA suggested that the proteins were indirectly involved during orthodontic treatment. An increased PA of HSPA1A and IGHV4–34 after 1 month of orthodontic treatment indicated a possible response to stress during the early phase of orthodontic treatment. HSPA1A was found to be associated with an apoptosis signalling pathway, a gonadotropin-releasing hormone receptor pathway, and Parkinson pathway (Fig. 3c). The acute inflammatory response has been shown to be involved during the early phase of orthodontic treatment, which may lead to apoptosis or cell death when induced by mechanical force [42]. Meanwhile, the increased PA of TYMP at T3 indicated growth promotion and angiogenic and chemotactic activity. TYMP has been found to have roles in maintaining the integrity of blood vessels, growth-promoting activity on endothelial cells, angiogenic activity in vivo, and chemotactic activity on endothelial cells in vitro [49]. A previous study reported that the production of angiogenic regulators in cultured human gingival and periodontal ligament fibroblasts was caused by mechanical stress [50].

IGKV3–20 showed increased PA after 6 months of orthodontic treatment, indicating that an immune response took place. VTN showed increased PA at T1 and T6, suggesting This that VTN was involved during inflammation and that it was affected by a mechanical force during the early phase of orthodontic treatment [51]. VTN is a cell adhesion and spreading factor found in serum and tissues. It interacts with glycosaminoglycans and proteoglycans [52]. It also acts as an inhibitor of the membrane-damaging effect of the terminal cytolytic complement pathway.

We found that there were significant differences in protein abundance pre-treatment and during orthodontic treatment. Of the proteins that showed differences in PA, only S100A9, IGJ, HSPA1A, IGHV4–34, TYMP, and VTN has the potential to be developed into potential early markers for root resorption, based on increases in PA during the early phase of orthodontic treatment. However, several limitations should be noted. Firstly, the sample size was small, and add to that the fact that the collected GCF samples were in a proportion of 9:1 female to male. Secondly, only three out of ten subjects were chosen randomly for further analyses using Orbitrap LC-MS/MS due to financial constraints. A total of 12 samples were collected at four time-points (T0, T1, T3 and T6) from three subjects. As all subjects have similar root resorption, the samples were selected randomly based on patient’s availability. With the current data, it was not possible to group based on low, medium, and high root resorption.

## Conclusions

Early detection of root resorption is extremely essential and may help orthodontists in patient management. The use of biomarkers is a better alternative to radiography as it will reduce unnecessary exposure to radiation. Our research has identified significant differences in protein abundance between pre-treatment and during orthodontic treatment. Out of these differences, six proteins - S100A9, IGJ, HSPA1A, IGHV4–34, TYMP, and VTN - showed significant increases in PA during the early phase of orthodontic tooth movement, making them prime candidates as potential biomarkers that can be exploited for detection of early root resorption.

## Data Availability

The datasets generated and analysed during the current study are available in the Figshare repository, https://figshare.com/s/ac7650ae247598fbc655.

## Abbreviations

AGC: Automatic gain control
AGT: Angiotensinogen
APOD: Apolipoprotein D
APOH: Beta-2-glycoprotein 1
AZGP1: Zinc-alpha-2-glycoprotein
BR: Biological regulation
CP: Cellular process
ELISA: Enzyme-linked immunosorbent assay
FDR: False discovery rate
GCF: Gingival crevicular fluid
HSPA1A: Heat shock 70 kDa protein 1A
HBB: Hemoglobin subunit beta
HRG: Histidine-rich glycoprotein
ICC: Intra-examiner correlation coefficient
IGHG1: Immunoglobulin heavy constant gamma 1
IGHV3–11: Immunoglobulin heavy variable 3–11
IGKV3–20: Immunoglobulin kappa variable 3–20
IGHV4–34: Immunoglobulin heavy variable 4–34
IGJ: Immunoglobulin J chain
ISP: Immune system process
ITMS: Ion trap mass spectrometry
kDa: Kilodalton
L: Localisation
LC: Liquid chromatography
LC-MS: Liquid chromatography-mass spectrometry
LC-MS/MS: Liquid chromatography-tandem mass spectrometry
min: Minute
mL: Milliliter
mM: Millimolar
mm: Millimeter
MP: Metabolic process
ms: Millisecond
MS2: Second stage mass spectrometry
MW: Molecular weight
m/z: Mass-to-charge ratio
ng: Nanogram
OTMS: Orbitrap mass spectrometry
PA: Protein abundance
PANTHER: Protein analysis through evolutionary relationships
pI: Isoelectric point
RS: Response to stimulus
S100A9: Protein S100-A9
STRING: Search tools or the retrieval of interesting proteins/genes
T0: Pre-treatment
T1: One month of orthodontic treatment
T3: Three months of orthodontic treatment
T6: Six months of orthodontic treatment
TIC: Total ion current
TMSB4X: Thymosin beta-4
TYMP: Thymidine phosphorylase
VTN: Vitronectin
×g: Times gravity
°C: Degree Celcius
μL: Microliter
%: Percent
™: Trademark
